# Increased Short-Term Variability of the QT Interval in Professional Soccer Players: Possible Implications for Arrhythmia Prediction

**DOI:** 10.1371/journal.pone.0018751

**Published:** 2011-04-15

**Authors:** Csaba Lengyel, Andrea Orosz, Péter Hegyi, Zsolt Komka, Anna Udvardy, Edit Bosnyák, Emese Trájer, Gábor Pavlik, Miklós Tóth, Tibor Wittmann, Julius Gy. Papp, András Varró, István Baczkó

**Affiliations:** 1 1st Department of Internal Medicine, Faculty of Medicine, University of Szeged, Szeged, Hungary; 2 Department of Pharmacology and Pharmacotherapy, University of Szeged, Szeged, Hungary; 3 Department of Health Sciences and Sports Medicine, Faculty of Physical Education and Sports Sciences, Semmelweis University, Budapest, Hungary; 4 Division of Cardiovascular Pharmacology, Hungarian Academy of Sciences, Szeged, Hungary; Tor Vergata University of Rome, Italy

## Abstract

**Background:**

Sudden cardiac death in competitive athletes is rare but it is significantly more frequent than in the normal population. The exact cause is seldom established and is mostly attributed to ventricular fibrillation. Myocardial hypertrophy and slow heart rate, both characteristic changes in top athletes in response to physical conditioning, could be associated with increased propensity for ventricular arrhythmias. We investigated conventional ECG parameters and temporal short-term beat-to-beat variability of repolarization (STV_QT_), a presumptive novel parameter for arrhythmia prediction, in professional soccer players.

**Methods:**

Five-minute 12-lead electrocardiograms were recorded from professional soccer players (n = 76, all males, age 22.0±0.61 years) and age-matched healthy volunteers who do not participate in competitive sports (n = 76, all males, age 22.0±0.54 years). The ECGs were digitized and evaluated off-line. The temporal instability of beat-to-beat heart rate and repolarization were characterized by the calculation of short-term variability of the RR and QT intervals.

**Results:**

Heart rate was significantly lower in professional soccer players at rest (61±1.2 vs. 72±1.5/min in controls). The QT interval was prolonged in players at rest (419±3.1 vs. 390±3.6 in controls, p<0.001). QTc was significantly longer in players compared to controls calculated with Fridericia and Hodges correction formulas. Importantly, STV_QT_ was significantly higher in players both at rest and immediately after the game compared to controls (4.8±0.14 and 4.3±0.14 vs. 3.5±0.10 ms, both p<0.001, respectively).

**Conclusions:**

STV_QT_ is significantly higher in professional soccer players compared to age-matched controls, however, further studies are needed to relate this finding to increased arrhythmia propensity in this population.

## Introduction

Sports activities are undoubtedly beneficial that improve quality of life and life expectancy, however, a number of tragic athletic field deaths have been reported in recent years, attracting widespread media attention. A significant amount of these cases involved elite professional soccer players [1]–[3]. Sudden death among young athletes is rare (1∶50 000–1∶100 000), however, it is still 2–4 times more frequent than in age-matched controls [4]. Although a number of congenital and acquired cardiac diseases have been identified to be in the background of sudden cardiac death in athletes (for a recent review see Pigozzi and Rizzo) [5], approximately 5% of SCD cases in athletes no structural abnormalities were detected in the heart upon autopsy, that is the heart appeared completely normal [6], [7]. The exact mechanism of SCD in these cases is not established and the cause is mostly attributed to ventricular fibrillation. In case of inconclusive autopsy findings, an ischemic origin of SCD is often suspected without hard evidence. In young athletes, SCD usually does not happen at peak performance, but during warmup, after training, or during a relatively inactive period of a competitive game, and ischemia specific signs on the ECG or proof of myocardial infarction is rarely found during or following these events. In addition, regular training is considered to lead to cardiac preconditioning, one of the most powerful cardioprotective (antiarrhythmic and antiischemic) mechanisms, that would significantly increase the chance for survival during these episodes [8], [9]. Therefore, as a cause myocardial ischemia in sudden cardiac death of young (<35 years) competitive athletes seems unlikely. Importantly, the scenario is quite different in older (>35 years) athletes, where ischemia is an important contributor to SCD, as reviewed by Pigozzi and Rizzo [5]. It should be noted that blunt trauma to the chest and concomitant cardiac contusion suffered during a game or training can also lead to electrocardiographic abnormalities [10] and sudden cardiac death [11].

Physical conditioning in competitive athletes induces cardiovascular adaptation including lower resting heart rate (increased vagal tone) and increased cardiac mass (hypertrophy) and volume as a consequence of increased demand on the cardiovascular system, called “athlete's heart”, a physiological compensatory mechanism that reverses in most cases following the termination of sports activities [12]. Echocardiography studies show that myocardial hypertrophy develops following long-term sports activities [12]–[14]. The largest increase in left ventricular cavity and wall thickness (>75%) was measured in cyclists, cross-country skiers, rowers, football players, and water polo players, while weight lifters, fencers, and wrestlers exhibited smaller changes (<50%) [15].

Myocardial hypertrophy in pathological settings in humans [16]–[18] and in animal models, especially in the chronic atrioventricular (AV) block dog model [19] and heart failure models [20]–[22], has been shown to cause electrophysiological remodeling where the expression of different ion channels, including potassium channels critical for repolarization, and exchangers is altered. In particular, the detected downregulation of different potassium channels (i.e. I_Ks_, I_Kr_ and I_K1_) in the chronic AV block dog model has been associated with increased incidence of serious ventricular arrhythmias probably due to decreased repolarization reserve [19]–[23].

Furthermore, the duration of repolarization is cycle length dependent and low heart rate in athletes leads to prolonged repolarization. These changes can also be associated with increased propensity for ventricular arrhythmias, including Torsades de Pointes (TdP). It is conceivable that prolonged repolarization, increased spatial dispersion of repolarization and a possibly impaired repolarization reserve due to myocardial hypertrophy-induced downregulation of potassium currents might represent increased risk for the development of ventricular arrhythmias, including TdP that can degenerate into VF and lead to sudden cardiac death in athletes.

In theory, if athletes with no apparent structural cardiac abnormalities but with increased susceptibility for cardiac arrhythmias could be identified, current screening methods could be improved to further decrease the incidence of sudden cardiac death in young athletes. However, current techniques for the reliable prediction of TdP and other, potentially fatal ventricular arrhythmias remain unsatisfactory. Based on recent evidence, in addition to the prolonged QTc interval, the short-term variability (STV) of repolarization can probably more reliably predict the development of TdP both in humans [24] and in animal models with decreased repolarization reserve [25], [26], and short-term variability of repolarization can increase when no noticable changes in the duration of cardiac repolarization are observed.

Since elevated STV of the QT interval (STV_QT_) has been associated with latent repolarization disorders and increased suspectibility to serious ventricular arrhythmias in LQT patients and patients with dilated cardiomyopathy [27], [28], the aim of this study was to compare conventional ECG parameters as well as the short-term beat-to-beat temporal variability of the RR and QT intervals of professional soccer players to age-matched controls who do not participate in competitive sports.

## Methods

### Ethics Statement

The studies described here were carried out in accordance with the Declaration of Helsinki (2000) of the World Medical Association and were approved by the Scientific and Research Ethical Committee of the Medical Scientific Board at the Hungarian Ministry of Health (ETT-TUKEB), under ethical approval No. 4987-0/2010-1018EKU (338/PI/010). All subjects have given written informed consent of the study.

### Study Subjects

The study population consisted of 76 male professional soccer players from the Hungarian Premier League (ages 16 to 39, mean 22.0±0.61 years; weight 76.2±0.95 kg, BMI 23.2±0.18 kg/m^2^), and 76 male, age-matched healthy control sedentary subjects who did not participate in sports activities (age 15 to 39, mean 22.0±0.54 years; weight 77±1.7 kg, BMI 23.3±0.48 kg/m^2^). Professional soccer players or age-matched controls were excluded from the study if they exhibited an excessive number (>5%) of ectopic atrial or ventricular beats, were in a rhythm other than normal sinus rhythm, had repolarization abnormalities (i.e. early repolarization pattern, T wave inversion and complete LBBB or RBBB), had a permanent pacemaker or had any other disorder such as serious retinopathy, symptomatic cardiac and/or pulmonary disease, acute metabolic disease, had excessive noise on the electrocardiographic signal that precluded analysis of the ECG waveform, were on any medication likely to affect the investigated parameters or consumed significant amount of food within 3 hours or drank alcohol, coffee or smoked within 10 hours. All of the control individuals and soccer players were of European descent.

### Electrocardiography

Five-minute 12-lead electrocardiograms (lead II) were recorded from professional soccer players and age-matched healthy human volunteers using Cardiosys H-01 software (Experimetria Ltd., Budapest, Hungary) in the supine position. The ECGs were digitized and stored on a computer for later analysis. The RR, QT intervals were measured using automated algorithms as the average of 30 beats, the frequency corrected QT interval (QTc) was calculated using Bazett's (QTc = QT/√RR), Fridericia (QTc = QT/[RR/1000]^1/3^), Framingham (QTc = QT+[0.154 * {1000-RR}]) and the Hodges formulas (QTc = QT+1.75 * [{60 000/RR}−60]).

In athletes, baseline ECG recordings were taken before a competitive soccer game (Hungarian Premier League) and also approximately 20 minutes after the end of the game in the dressing room.

The calculation of the short-term beat-to-beat variability of repolarization was chosen since it is a relatively simple method that has been suggested as a future screening tool; moreover it has been shown in animal studies [25], [26] and in certain patient populations [27], [28] to reliably predict increased arrhythmia propensity.

Using 30 consecutive beats, RR and QT intervals were plotted against their respective previous interval and Poincaré plots were constructed as described previously [23]. The instability of beat-to-beat heart rate and repolarization were characterized by the short-term variability (STV) of the RR and QT intervals, and were calculated using the following formula: STV = ∑|D_n+1_−D_n_| (30×√2)^−1^, where D is the duration of the RR or QT intervals. This calculation defines the STV as the mean distance of points perpendicular to the line of identity in the Poincaré plot.

### Echocardiography

Echocardiographic measurements were performed at rest on 23 professional soccer players and 23 age-matched controls using a Dornier AI 4800 (Germany) echocardiograph with a 2.5 MHz transducer. Two-dimensionally guided M-mode recordings were obtained parasternally in accordance with the recommendations of the American Society of Echocardiography [29]. Measurements were carried out as described previously [30]. For purely logistic reasons, not all control individuals and soccer players were subjected to echocardiography.

### Statistics

Body weight, BMI, age and ECG interval data are expressed as means ± standard error of the mean (S.E.M.). Comparisons between controls and soccer players were made using the unpaired Student's *t*-test. ECG parameters of athletes before and after the game were compared by one-way analysis of variance (ANOVA) followed by a paired *t*-test. A *p* value of <0.05 was considered significantly different. Statistical analyses were performed using Statistica for Windows (version 9).

## Results

### Echocardiography measurements in study subjects

Professional soccer players exhibited significantly higher values in interventricular septum, left ventricular posterior wall thickness and in left ventricular internal diameter during diastole compared to age-matched controls (Table 1). These results were not unexpected and were supportive of the presence of athlete's heart in these professional soccer players.

**Table 1:** Echocardiographic parameters in professional soccer players and age matched controls.

	IVSd (mm)	LVPWd (mm)	LVIDd (mm)	LVIDs (mm)
**Controls**	9.0±0.31	9.1±0.9	48.1±0.95	31.9±0.96
**Soccer players**	10.2±0.20**	9.9±0.14**	50.6±0.80*	33.3±0.66

IVSd: interventricular septum thickness during diastole; LVPWd: left ventricular posterior wall thickness; LVIDd, LVIDs: left ventricular internal diameter during diastole and systole; n = 23 in each group,

*p<0.05,

**p<0.01 vs. control.

### Heart rate, QT and QTc intervals in study subjects

**Figure 1:**
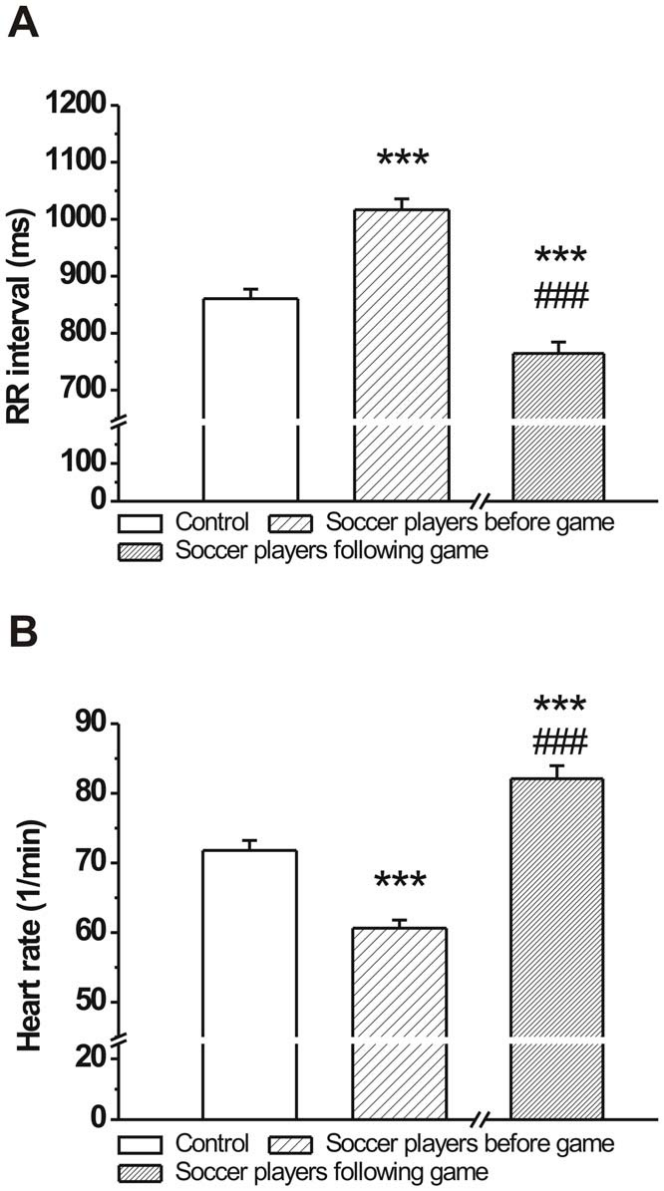
The RR interval and heart rate of age-matched controls and professional soccer players before and following a competitive game. **A**: The RR interval was significantly longer (**A**) and heart rate was significantly lower (**B**) in soccer players before the game compared to controls (n = 76 persons/group; ***p<0.001 vs. age-matched control; Means ± S.E.M.; ^###^p<0.001 vs. before game values).

The development of athlete's heart in response to long-term physical conditioning is characterized by increased vagal tone. As expected, the RR intervals in soccer players were significantly longer before the game compared to age-matched volunteers (Fig. 1A). Consequently, the heart rate of professional soccer players were lower compared to the control group before the game (Fig. 1B). However, after the soccer game the heart rates of athletes were higher than in controls (Fig. 1B).

**Figure 2:**
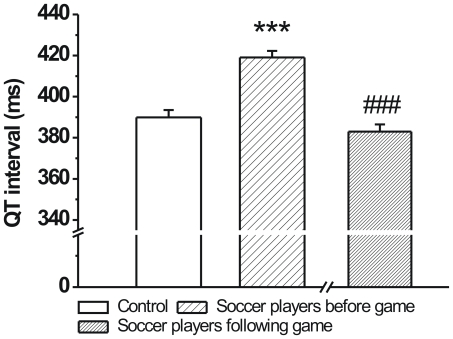
The QT intervals of age-matched controls and professional soccer players before and following a competitive game. The QT interval was significantly longer in soccer players before the game (n = 76 persons/group; ***p<0.001 vs. age-matched control; Means ± S.E.M.; ^###^p<0.001 vs. before game values).

The duration of cardiac repolarization is cycle length dependent where slower heart rates lead to prolonged repolarization. Accordingly, significantly longer QT intervals and were measured in soccer players before the game (Fig. 2). However, after the game these differences in QT intervals were not observed, since heart rates of athletes were similar to controls, while the QT intervals in soccer players were significantly shorter than before the game (Fig. 2.).

**Figure 3:**
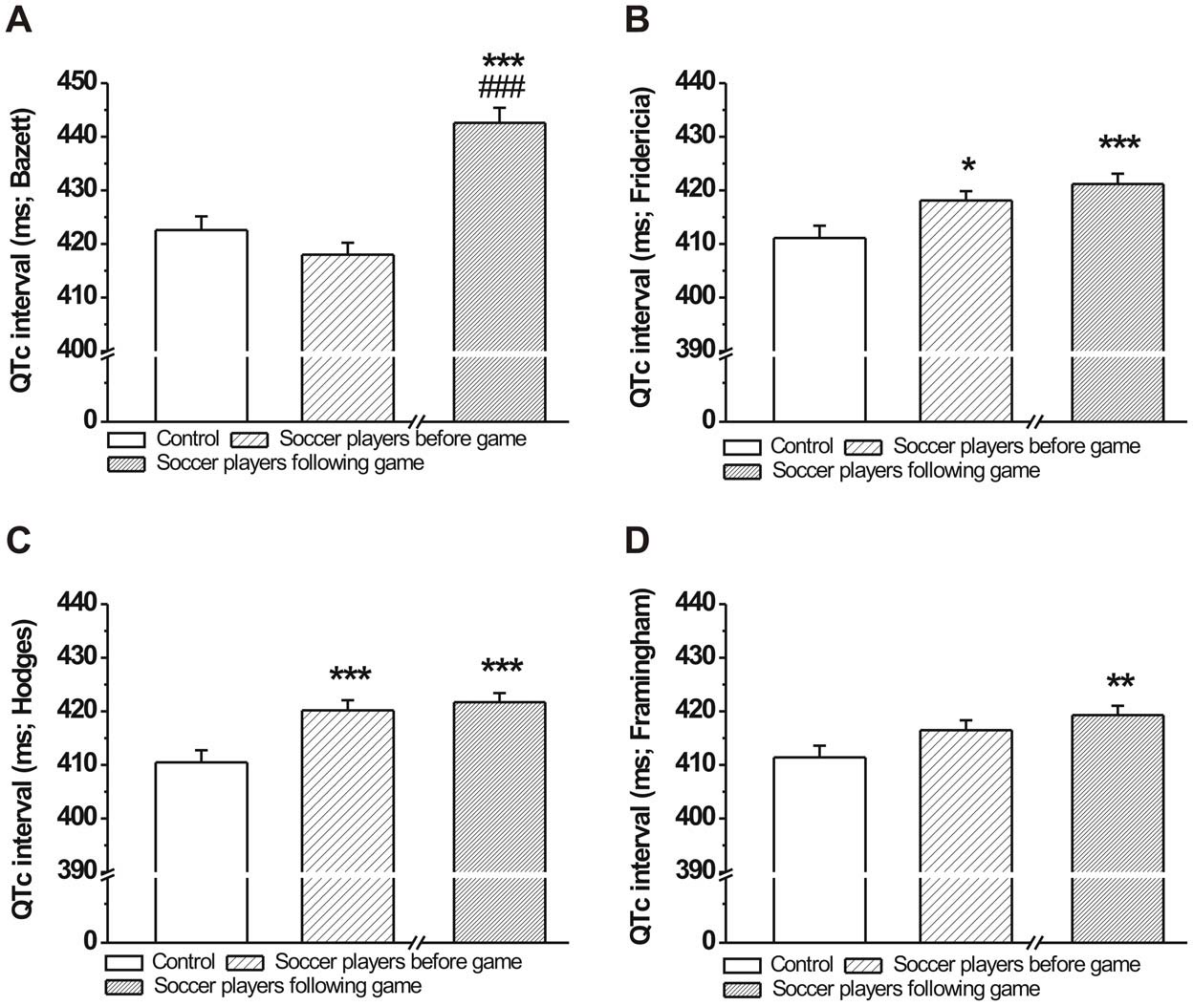
Frequency corrected QT interval of age-matched controls and professional soccer players before and following a competitive game. QTc interval calculated with the Bazett formula was not different in soccer players before the game and was significantly prolonged after the game (**A**). QTc values calculated with the Fridericia and Hodges formulae but not the Framingham formula showed significant difference between groups before the game, and none of the three calculations yielded any difference between before and after game values in soccer players (**B**, **C** and **D**; n = 76 persons/group; Means ± S.E.M.; *p<0.05; **p<0.01; ***p<0.001 vs. age-matched control; ^###^p<0.001 vs. before game values).

In order to reliably assess the duration of ventricular repolarization and to minimize the influence of changing heart rate on the QT interval, it is necessary to carry out frequency correction of the QT interval. However, recent work has shown that Bazett and Fridericia correction formulas described over 90 years ago [31], [32] overestimate changes in QT interval [33]. Therefore, in this study, to calculate the frequency corrected QT interval (QTc) we also used Framingham and Hodges correction formulas shown to alter the accuracy of QT interval changes due to heart rate in a smaller degree [33]. In this regard, QTc calculated using the Bazett and Framingham formulae were not different in athletes before the game compared to controls (Fig. 3A and D), while QTc values calculated with the other two formulas were significantly longer in players before the game (Fig. 3B and D). In addition, QTc was significantly prolonged in soccer players following the game compared to control values calculated with all correction formulae in the present study (Fig. 3A–D). Only QTc calculated with the Bazett formula showed a large and significant prolongation in soccer players after the game compared to pre-game values (Fig. 3A–D).

### Short term beat-to-beat variability of the RR and QT intervals

**Figure 4:**
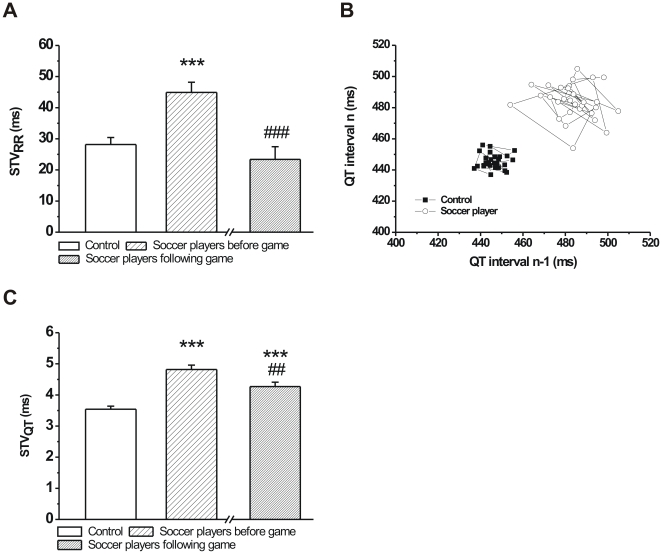
Short-term beat-to-beat temporal variability of the RR (STV_RR_) and QT (STV_QT_) intervals in age-matched controls and professional soccer players before and following a competitive game. Soccer players had a significantly higher STV_RR_ compared to controls before the game. STV_RR_ was similar to controls in soccer players immediately after the game (**A**). Poincaré plots illustrating short-term temporal variability of the QT interval at rest in a control individual and in a professional soccer player before the game. Note the shift of QT values to the right and upwards in the soccer player indicating QT prolongation and the increased scattering of QT interval values in the soccer player demonstrating increased beat-to-beat variability of the QT interval (**B**). Short-term QT variability was significantly higher in soccer players both before and after the game compared to controls but also decreased in players compared to pre-game values (**C**). (n = 76 persons/group; Means ± S.E.M.; ***p<0.001 vs. age-matched control; ^##^p<0.01; ^###^p<0.001 vs. before game values).

To characterize the instability of cardiac ventricular repolarization, the short-term beat-to-beat variability of the QT interval was calculated in professional soccer players and age-matched controls. Since it is reasonable to assume that STV_QT_ can be, at least in part, influenced by the short-term variability of the RR interval, the STV_RR_ was also calculated in both groups. Soccer players before the competitive game exhibited a significantly larger STV_RR_ compared to controls, however, this difference disappeared after the game, when their heart rates were similar to controls (Fig. 4A).

**Figure 5:**
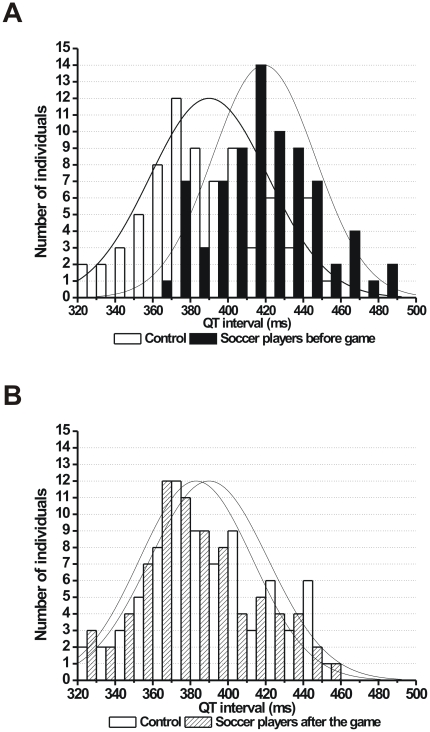
Histograms showing the distribution of the QT interval. (**A**) Controls (empty bars) and soccer players before game (full bars) and (**B**) controls (empty bars) and soccer players after the game (hashed bars). Bin size is 10 ms. (n = 76 persons/group).

**Figure 6:**
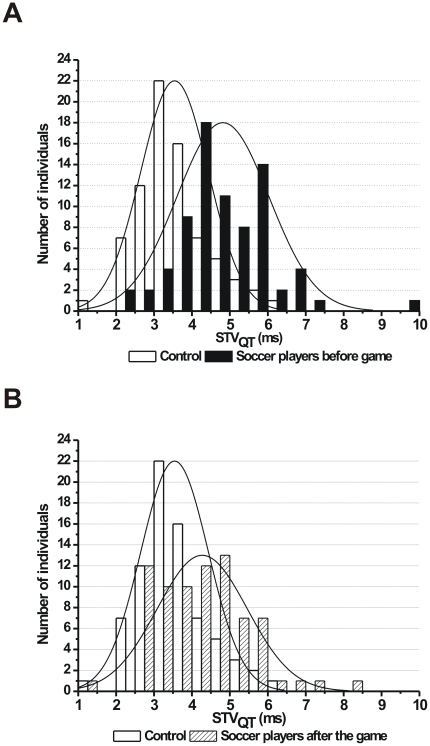
Histograms showing the distribution of short-term beat-to-beat variability of the QT interval (STV_QT_). (**A**) Controls (empty bars) and soccer players before game (full bars) and (**B**) controls (empty bars) and soccer players after the game (hashed bars). Bin size is 0.5 ms. (n = 76 persons/group).

As individual representative examples (Poincaré plots) and grouped average data show, the short-term beat-to-beat variability of the QT interval was significantly higher in soccer players compared to controls (Fig. 4B and C). Importantly, and unlike the STV_RR_, the STV_QT_ was still significantly higher in soccer players compared to controls but was also reduced after the game compared to pre-game values (Fig. 4C). Histograms on Figures 5 and 6 show the distribution of QT interval and STV_QT_ values within the control and soccer player groups, respectively. The histograms clearly exhibit a shift to the right in the distribution of both QT intervals and STV_QT_ in soccer players before the competitive game compared to controls (Figs. 5A and 6A). However, while the distribution of QT intervals show a similar pattern in soccer players to controls following the game (Fig. 5B), the distribution pattern of STV_QT_ largely remained unchanged after the game (Fig. 6B). These results suggest that the increased STV_QT_ in soccer players is very unlikely caused by the prolonged QT interval itself in these athletes.

In six players before the game the STV_QT_ was markedly larger than the average STV_QT_ in the soccer player group (9.7, 7.2, 7.0, 7.0, 6.7, 6.7 and group average was 4.8±0.14 ms; Fig. 6.). Since increased STV_QT_ has been associated with increased proarrhythmic risk in certain patient populations, the player who had 9.7 ms STV_QT_ was contacted and the measurement was repeated to yield a heart rate of 47/min and an STV_QT_ of 5.0 ms, however, before the repeated measurement he had been injured for 2 months. Whether this smaller (but still higher and close to the group average) STV_QT_ on repeated measurement was due to the well-known de-training phenomenon in an athlete who had been out of training due to injury was unclear.

## Discussion

The main and novel finding of this study is that short-term beat-to-beat variability of the QT interval is significantly increased in professional soccer players compared to age-matched healthy volunteers. The increased STV_QT_ was accompanied by a prolonged QT, and a lengthened Fridericia and Hodges QT_c_ interval in these athletes.

In competitive athletes, the cardiovascular system adapts to chronic physical exercise by the development of “athlete's heart”, characterized by lower resting heart rate (increased vagal tone), increased ventricular mass (hypertrophy) and volume to meet the increased demand.

In a reasonable animal experimental analogue for athlete's heart, in dogs with chronic AV block, myocardial hypertrophy and downregulation of potassium channels, most notably of the slow component of the delayed rectifier potassium current (I_Ks_), develops [34]. These animals are more susceptible to lethal ventricular arrhythmias subjected to various challenges [19]. I_Ks_ has been identified as a key component in the somewhat redundant repolarizing capacity of the myocardium, termed repolarization reserve [35], [36]. Repolarization reserve refers to the heart's compensating ability for loss or impaired function of one or more potassium currents critical for normal repolarization [37]. Impaired repolarization reserve does not necessarily lead to clinically manifest repolarization abnormalities on the ECG but makes the heart more susceptible to arrhythmia development [37]–[39]. The downregulation of repolarizing potassium currents, including the I_K1_, I_to_, I_Kr_ and I_Ks_ has also been shown both in animal models and patients with heart failure, leading to prolonged repolarization, increased dispersion of repolarization with concomitant increase in the incidence of serious ventricular arrhythmias [36], [40]–[42]. It might be plausible that myocardial hypertrophy, whatever the underlying cause, may lead to potassium channel downregulation and may result in decreased repolarization reserve and increased propensity for arrhythmias including Torsades de Pointes, a characteristic arrhythmia that can degenerate into ventricular fibrillation and culminate in sudden cardiac death. It should be noted that autonomic and cardiac electrophysiological changes in dogs with chronic AV block possibly do not exactly mirror those developing in athlete's heart, however, few animal experimental data are available on the effect of endurance exercise training on cardiac hypertrophy and electrophysiology in species that are highly relevant to human, i.e. not in mice and rats. Some of these studies observed slowed heart rate, prolonged QT interval and ECG signs of cardiac hypertrophy in such animals [43], [44]. Whether a ventricular electrical remodeling leading to decreased repolarization reserve develops in these animals is not known, however, it has been speculated that in top endurance athletes, downregulation of potassium channels might occur [45].

There are two main prerequisites for the development of TdP chaotic ventricular tachycardia and consequent fatal ventricular fibrillation: an arrhythmia substrate (prolonged repolarization, spatial and temporal inhomogeneity of repolarization creating re-entry paths) and a trigger (extrasystole in the vulnerable period) for the initiation of the arrhythmic event. Increased vagal tone in athletes lowers heart rate that favors prolonged repolarization and increased inhomogeneity. The possible potassium channel downregulation due to myocardial hypertrophy also prolongs repolarization and reduces repolarization reserve. In some athletes, loss of function mutations of repolarizing potassium channels and/or gain of function mutations of sodium channels may be present. In these individuals with impaired repolarization reserve, additional, most likely moderate potassium channel blocking effects can provoke TdP arrhythmias that, in some cases lead to ventricular fibrillation. Theoretically, in this scenario a number of conditions, compounds and dietary constituents can precipitate such events of sudden cardiac death (as recently reviewed by Varró and Baczkó) [46]. These may include serum electrolyte changes (e.g. hypokalemia when fluid intake is not adequate), food and drinks containing flavonoids with HERG inhibitory effects [47], medications with various degree of HERG and other potassium channel blocking properties. In this regard, the celecoxib has been shown to block Kv2.1 channels [48]. Non-steroid antiinflammatory drugs are used by athletes very often and in large doses to treat sports injuries. These factors can create and enhance the arrhythmia substrate in athletes, while elevated intracellular cAMP levels due to increased sympathetic disharge may contribute to trigger extrasystole generation via increased pacemaker (I_f_) current [49] and/or increased L-type calcium current [50].

The reliable identification of patients at risk for serious ventricular arrhythmia and sudden cardiac death remains elusive. Accumulating evidence suggests that QT interval prolongation alone cannot reliably predict the development of TdP since cardiac repolarization reserve may be reduced without siginificant changes in the duration of cardiac repolarization. A number of clinical studies [27], [28], [51] and data from *in vivo* animal experiments using species that are electrophysiologically relevant for humans in regard of ventricular repolarization [26], [52]–[54] as well as *in vitro* studies [55], [56] strongly suggest that the short-term variability of the duration of repolarization (i.e. QT interval on the ECG) may be a better novel parameter to predict serious ventricular arrhythmias. These studies found that increased STV_QT_ correlated with elevated incidence of lethal ventricular arrhythmias and sudden cardiac death. Importantly, the patients and experimental animals in all of these studies had narrowed repolarization reserve, albeit due to different mechanisms, ranging from pharmacological inhibition of repolarizing potassium channels to downregulation of potassium currents during electrical remodeling and including mutations in ion channels leading to congenital long QT syndromes. Therefore, based on these studies and the present results, the elevated temporal beat-to-beat variability in competitive soccer players may indicate a larger repolarization instability and an increased propensity for ventricular arrhythmias. Notably, physical deconditioning in trained athletes with no cardiac structural abnormalities decreased the incidence and complexity of ventricular tachyarrhytmias [57].

### Study limitations

For purely practical and logistic reasons echocardiographic assessment of all soccer players and all controls were not performed in this study. However, our echocardiography data randomly performed on 23 soccer players and 23 controls support the findings of a number of previous studies showing that endurance athletes, including soccer players, as part of the cardiovascular system's physiological response to long-term intense physical training, develop athlete's heart that features myocardial hypertrophy [12]–[14], [58], [59]. In our study, professional soccer players from the first division were enlisted who participated in rigorous endurance training schedule for years based on international standards. We also found they had significantly decreased resting heart rate and consequent prolongation of the QT interval, both are characteristics of athlete's heart. Based on the above it is assumed that the echocardiography data are representative for their respective groups.

Since the duration of repolarization is cycle length dependent, variability in the RR interval could influence QT variability. Based on our results, the influence of STV_RR_ on STV_QT_ cannot be ruled out, however, in professional soccer players STV_RR_ was reduced and was similar to control values after the competitive game while STV_QT_ remained significantly higher, strongly suggesting that STV_QT_ was increased irrespective of changes in STV_RR_. The short-term variability of the monophasic action potential was found to be partially influenced by pacing cycle length and was moderately decreased at faster cycle lengths in anesthetized dogs with chronic AV block characterized by marked bradycardia and myocardial hypertrophy [52].

We found elevated STV_QT_ in the present study in soccer players, who were chosen as subjects of the study since the different degrees of cardiac hypertrophy found in athletes of different sports [15] may have significant influence on cardiac repolarization, and sudden cardiac death associated with sports activity has been reported most commonly in Europe among soccer players [60]. However, the changes in STV_QT_ can be different in other sports, depending on type, intensity and duration of various training schedules used in different sports. Further studies are needed to confirm whether STV_QT_ elevation is uniformly present in other endurance athletes.

### Conclusions

In conclusion, the short-term variability of the QT interval is elevated in professional soccer players, which, according to our present knowledge, might indicate increased repolarization instability even without any underlying cardiac disease. Based on the available literature, decreased repolarization reserve due to downregulation of certain repolarizing potassium currents associated with myocardial hypertrophy may underlie these changes. As clinical and animal studies illustrate, increased STV_QT_ may be more predictive for the development of serious ventricular arrhythmias than conventional ECG parameters, such as the prolongation of the QT_c_ interval. In our study, some soccer players exhibited greatly increased STV_QT_ even when compared to other players, suggesting that it may be beneficial to screen athletes for elevated repolarization instability by adding the relatively low cost STV_QT_ determination to routine ECG examinations. Individual athletes with large STV_QT_ could be then subjected to more detailed and sophisticated examinations (e.g. evaluation of possible mutations in potassium channel protein encoding genes) to carefully evaluate their vulnerability to ventricular arrhythmias and sudden cardiac death. Importantly, our results further support the inclusion of ECG in preparticipation athlete screening expertly worked out by Corrado *et al.* [61], with the notion that calculation of STV_QT_ could also be added to the ECG evaluation in case our findings can be confirmed in a broader athlete population. It is important to emphasize that no arrhythmias were observed among soccer players in this study and further, more comprehensive investigations are needed to establish whether the higher STV_QT_ relates to higher arrhythmia propensity in this population. This study also warrants the investigation of STV_QT_ in top athletes with various training levels and in a larger number of athletes preferably taking part in different types of sports activities to enable investigators to make a direct link between STV_QT_, arrhythmia susceptibility and sudden cardiac death in top competitive athletes.

## References

[1] Cameroon star Foe dies: Cameroon midfielder Marc-Vivien Foe dies after collapsing during an international match in France.. http://news.bbc.co.uk/sport1/hi/football/3024360.stm.

[2] Benfica striker dies.. http://news.bbc.co.uk/sport2/hi/football/europe/3428803.stm.

[3] Motherwell captain O'Donnell dies.. http://news.bbc.co.uk/sport2/hi/football/teams/m/motherwell/7164150.stm.

[4] Corrado D, Michieli P, Basso C, Schiavon M, Thiene G (2007). How to screen athletes for cardiovascular diseases.. Cardiol Clin.

[5] Pigozzi F, Rizzo M (2008). Sudden death in competitive athletes.. Clin Sports Med.

[6] Maron BJ, Roberts WC, McAllister HA, Rosing DR, Epstein SE (1980). Sudden death in young athletes.. Circulation.

[7] Maron BJ, Shirani J, Poliac LC, Mathenge R, Roberts WC (1996). Sudden death in young competitive athletes. Clinical, demographic, and pathological profiles.. JAMA.

[8] Parratt JR, Végh Á (1997). Delayed protection against ventricular arrhythmias by cardiac pacing.. Heart.

[9] Kavazis AN (2009). Exercise preconditioning of the myocardium.. Sports Med.

[10] Vago H, Toth A, Apor A, Maurovich-Horvat P, Toth M (2010). Images in cardiovascular medicine. Cardiac contusion in a professional soccer player: visualization of acute and late pathological changes in the myocardium with magnetic resonance imaging.. Circulation.

[11] Madias C, Maron BJ, Weinstock J, Estes NA, Link MS (2007). Commotio cordis - sudden cardiac death with chest wall impact.. J Cardiovasc Electrophysiol.

[12] Atchley AE, Douglas PS (2007). Left ventricular hypertrophy in athletes: morphologic features and clinical correlates.. Cardiol Clin.

[13] Paolo FM, Pelliccia A The “Athlete's heart”: relation to gender and race.. Cardiol Clin.

[14] Scharhag J, Schneider G, Urhausen A, Rochette V, Kramann B (2002). Athlete's heart: right and left ventricular mass and function in male endurance athletes and untrained individuals determined by magnetic resonance imaging.. J Am Coll Cardiol.

[15] Maron BJ, Pelliccia A (2006). The heart of trained athletes: cardiac remodeling and the risks of sports, including sudden death.. Circulation.

[16] Janse MJ (2004). Electrophysiological changes in heart failure and their relationship to arrhythmogenesis.. Cardiovasc Res.

[17] Li GR, Lau CP, Leung TK, Nattel S (2004). Ionic current abnormalities associated with prolonged action potentials in cardiomyocytes from diseased human right ventricles.. Heart Rhythm.

[18] Nattel S, Maguy A, Le Bouter S, Yeh YH (2007). Arrhythmogenic ion-channel remodelling in the heart: heart failure, myocardial infarction, and atrial fibrillation.. Physiol Rev.

[19] Vos MA, de Groot SH, Verduyn SC, van der Zande J, Leunissen HD (1998). Enhanced susceptibility for acquired torsade de pointes arrhythmias in the dog with chronic, complete AV block is related to cardiac hypertrophy and electrical remodeling.. Circulation.

[20] Li GR, Lau CP, Ducharme A, Tardif JC, Nattel S (2002). Transmural action potential and ionic current remodelling in ventricles of failing canine hearts.. Am J Physiol Heart Circ Physiol.

[21] Nuss HB, Kaab S, Kass DA, Tomaselli GF, Marbán E (1999). Cellular basis of ventricular arrhythmias and abnormal automaticity in heart failure.. Am J Physiol Heart Circ Physiol.

[22] Rose J, Armoundas AA, Tian Y, DiSilvestre D, Burysek M (2005). Molecular correlates of altered expression of potassium currents in failing rabbit myocardium.. Am J Physiol Heart Circ Physiol.

[23] Volders PG, Sipido KR, Vos MA, Spätjens RL, Leunissen JD (1999). Downregulation of delayed rectifier K^+^ currents in dogs with chronic complete atrioventricular block and acquired torsades de pointes.. Circulation.

[24] Haigney MC, Zareba W, Gentlesk PJ, Goldstein RE, Illovsky M (2004). QT interval variability and spontaneous ventricular tachycardia or fibrillation in the multicenter automatic defibrillator implantation trial (MADIT) II patients.. J Am Coll Cardiol.

[25] Thomsen MB, Verduyn SC, Stengl M, Beekman JD, de Pater G (2004). Increased short-term variability of repolarization predicts d-sotalol-induced torsades de pointes in dogs.. Circulation.

[26] Lengyel Cs, Varró A, Tábori K, Papp JG, Baczkó I (2007). Combined pharmacological block of I_Kr_ and I_Ks_ increases short-term QT interval variability and provokes torsades de pointes.. Brit J Pharmacol.

[27] Hinterseer M, Beckmann BM, Thomsen MB, Pfeufer A, Dalla Pozza R (2009). Relation of increased short-term variability of QT interval to congenital long-QT syndrome.. Am J Cardiol.

[28] Hinterseer M, Beckmann BM, Thomsen MB, Pfeufer A, Ulbrich M (2010). Usefulness of short-term variability of QT intervals as a predictor for electrical remodeling and proarrhythmia in patients with nonischemic heart failure.. Am J Cardiol.

[29] Sahn DJ, De Maria A, Kisslo J, Weyman A (1978). Recommendations regarding quantitation in M-mode echocardiography: results of a survey of echocardiographic measurements.. Circulation.

[30] Pavlik G, Olexó Zs, Frenkl R (1996). Echocardiographic estimates related to various body size measures in athletes.. Acta Physiol Hung.

[31] Bazett H (1920). An analysis of the time relationship of electrocardiograms.. Heart.

[32] Fridericia LS (1920). Die Systolendauer im elektrokardiogramm bei normalen menschen und bei herzkranken.. Acta Med Scand.

[33] Indik JH, Pearson EC, Fried K, Woosley RL (2006). Bazett and QT correction formulas interfere with measurement of drug-induced changes in QT interval.. Heart Rhythm.

[34] Volders PG, Sipido KR, Vos MA, Spätjens RL, Leunissen JD (1999). Downregulation of delayed rectifier K^+^ currents in dogs with chronic complete atrioventricular block and acquired torsades de pointes.. Circulation.

[35] Varró A, Baláti B, Iost N, Takács J, Virág L (2000). The role of the delayed rectifier component I_Ks_ in dog ventricular muscle and Purkinje fibre repolarization.. J Physiol.

[36] Jost N, Papp JG, Varró A (2007). Slow delayed rectifier potassium current (I_Ks_) and the repolarization reserve.. Ann Noninvasive Electrocardiol.

[37] Roden DM (1998). Taking the idio out of idiosyncratic – predicting torsades de pointes.. Pacing Clin Electrophysiol.

[38] Roden DM, Yang T (2005). Protecting the heart against arrhythmias: potassium current physiology and repolarization reserve.. Circulation.

[39] Varró A, Papp JG (2006). Low penetrance, subclinical congenital LQTS: concealed LQTS or silent LQTS?. Cardiovasc Res.

[40] Tomaselli GF, Beuckelmann DJ, Calkins HG, Berger RD, Kessler PD (1994). Sudden cardiac death in heart failure: the role of abnormal repolarization.. Circulation.

[41] Akar FG, Rosenbaum DS (2003). Transmural electrophysiological heterogeneities underlying arrhythmogenesis in heart failure.. Circ Res.

[42] Tamargo J, Caballero R, Gómez R, Valenzuela C, Delpón E (2004). Pharmacology of cardiac potassium channels.. Cardiovasc Res.

[43] Constable PD, Hinchcliff KW, Olson J, Hamlin RL (1994). Athletic heart syndrome in dogs competing in a long-distance sled race.. J Appl Physiol.

[44] Constable PD, Hinchcliff KW, Olson JL, Stepien RL (2000). Effects of endurance training on standard and signal-averaged electrocardiograms of sled dogs.. Am J Vet Res.

[45] Hart G (2003). Exercise-induced cardiac hypertrophy: a substrate for sudden death in athletes?. Exp Physiol.

[46] Varró A, Baczkó I (2010). Possible mechanisms of sudden cardiac death in top athletes: a basic cardiac electrophysiological point of view.. Pflüg Arch Eur J Physiol.

[47] Zitron E, Scholz E, Owen RW, Lück S, Kiesecker C (2005). QTc prolongation by grapefruit juice and its potential pharmacological basis. HERG channel blockade by flavonoids.. Circulation.

[48] Frolov RV, Berim IG, Singh S (2008). Inhibition of delayed rectifier potassium channels and induction of arrhythmia: a novel effect of celecoxib and the mechanism underlying it.. J Biol Chem.

[49] DiFrancesco D, Borer JS (2007). The funny current: cellular basis for the control of heart rate.. Drugs.

[50] Sperelakis N, Katsube Y, Yokoshiki H, Sada H, Sumii K (1996). Regulation of the slow Ca^++^ channels of myocardial cells.. Mol Cell Biochem.

[51] Hinterseer M, Thomsen MB, Beckmann BM, Pfeufer A, Schimpf R (2008). Beat-to-beat variability of QT intervals is increased in patients with drug-induced long-QT syndrome: a case control pilot study.. Eur Heart J.

[52] Thomsen MB, Truin M, van Opstal JM, Beekman JD, Volders PG (2005). Sudden cardiac death in dogs with remodeled hearts is associated with larger beat-to-beat variability of repolarization.. Basic Res Cardiol.

[53] Thomsen MB, Oros A, Schoenmakers M, van Opstal JM, Maas JN (2007). Proarrhythmic electrical remodelling is associated with increased beat-to-beat variability of repolarisation.. Cardiovasc Res.

[54] Hanton G, Yvon A, Racaud A (2008). Temporal variability of QT interval and changes in T wave morphology in dogs as markers of the clinical risk of drug-induced proarrhythmia.. J Pharmacol Toxicol Methods.

[55] So PP, Backx PH, Dorian P (2008). Slow delayed rectifier K^+^ current block by HMR 1556 increases dispersion of repolarization and promotes Torsades de Pointes in rabbit ventricles.. Brit J Pharmacol.

[56] Abi-Gerges N, Valentin JP, Pollard CE (2010). Dog left ventricular midmyocardial myocytes for assessment of drug-induced delayed repolarization: short-term variability and proarrhythmic potential.. Brit J Pharmacol.

[57] Biffi A, Maron BJ, Verdile L, Fernando F, Spataro A (2004). Impact of physical deconditioning on ventricular tachyarrhythmias in trained athletes.. J Am Coll Cardiol.

[58] Shapiro LM (1984). Physiological left ventricular hypertrophy.. Brit Heart J.

[59] Abernethy WB, Choo JK, Hutter AM (2003). Echocardiographic characteristics of professional football players.. J Am Coll Cardiol.

[60] Corrado D, Basso C, Schiavon M, Thiene G (1998). Screening for hypertrophic cardiomyopathy in young athletes.. N Eng J Med.

[61] Corrado D, Basso C, Schiavon M, Pelliccia A, Thiene G (2008). Pre-participation screening of young competitive athletes for prevention of sudden cardiac death.. J Am Coll Cardiol.

